# O8.6. THE RELATIONSHIP BETWEEN COGNITION AND FUNCTIONAL IMPROVEMENT IN THE CONTEXT OF A PSYCHOSOCIAL INTERVENTION TARGETING SOCIAL DISABILITY IN FIRST EPISODE PSYCHOSIS

**DOI:** 10.1093/schbul/sby015.242

**Published:** 2018-04-01

**Authors:** Sian Griffiths, Stephen Wood, Renate Reniers, Max Birchwood

**Affiliations:** 1 University of Birmingham; 2 Orygen, the National Centre of Excellence in Youth Mental Health; 3 Warwick

## Abstract

**Background:**

Whilst Early Intervention Services (EIS) are the ‘gold standard’ treatment for young people with psychosis, in a recent study of over 1000 First Episode Psychosis (FEP) cases, 66% of individuals were experiencing a high level of poor functioning, despite receiving care under EIS for a period of 12 months (Hodgekins et al., 2015). This highlights the need to develop new interventions to target functional impairments in FEP.

A specialised Social Recovery Cognitive Behavioural Therapy (SRCBT) has been developed which aims to address the underlying factors impeding social recovery, and has shown to be effective at improving structured activity in individuals with established illness and FEP (Fowler et al., 2013).

Identifying the factors that contribute to functional change will ensure that targeted psychosocial therapies are being delivered appropriately. Impaired social cognition (SC) and neurocognition (NC) are closely related to poor functioning in psychosis. Exploration of SC and NC pre- and post-intervention will therefore be important to test underlying mechanisms of functional change, and identify individuals who are more likely to benefit from the specialized SRCBT.

**Methods:**

This study ran alongside a multi-site proof of concept trial of SRCBT, for individuals with FEP experiencing social disability. Participants (M age = 25 years) had less than 30 hours a week of structured activity before entering the trial. At baseline, 123 participants completed a battery of SC and NC assessments. 59 participants were randomly allocated to the therapy group (SRCBT + EIS), and 64 were randomly allocated to the standard care group (care from an EIS alone). Participants completed a follow-up assessment at 9 months on the same cognitive battery, and a further assessment of their structured activity. The assessors were blind to group allocation. A small sub-sample of participants (N=6) allocated to the SRCBT group underwent functional magnetic resonance imaging (fMRI) scanning pre- and post- SRCBT, to explore any changes in the social brain regions following successful intervention.

**Results:**

Regression analyses showed that SC was a significant predictor of treatment response (i.e. improved structured activity). Specifically, those who had better social knowledge at baseline were most likely to benefit from the SRCBT (Wald χ^2^ = 4.073; p = .044), accounting for 16% of the overall variance. To further illustrate this, individuals scoring in the top quartile for social knowledge achieved an additional 11 hours on average of structured activity post-intervention.

Furthermore, in the group that underwent fMRI scanning pre- and post - intervention, there were increased activations in the social brain regions, namely the temporo-parietal junction (TPJ), which became more refined and localized by follow-up. There was also a trend for increased signal intensity in the TPJ, with increased structured activity post-SRCBT. Although this was not significant (r = .455; p = .365), there was a moderate strength relationship

**Discussion:**

No studies to-date have examined predictors of treatment response to a CBT intervention targeting functional impairment in FEP. These findings have implications for practice where remediation of SC may improve the efficacy of the SRCBT, particularly for individuals who have poorer social knowledge. This study is also the first to provide preliminary insights into a functional brain network associated with improved structured activity in psychosis; however, replication of these findings in a larger sample is needed.

